# Contrasting demographic history and gene flow patterns of two mangrove species on either side of the Central American Isthmus

**DOI:** 10.1002/ece3.1569

**Published:** 2015-07-28

**Authors:** Ivania Cerón-Souza, Elena G Gonzalez, Andrea E Schwarzbach, Dayana E Salas-Leiva, Elsie Rivera-Ocasio, Nelson Toro-Perea, Eldredge Bermingham, W Owen McMillan

**Affiliations:** 1 Smithsonian Tropical Research InstituteApartado, 0843-03092, Panama; 2 University of Puerto Rico – Rio Piedras CampusPO BOX 23360, San Juan, Puerto Rico, 00931-3360; 3 Departamento de Biodiversidad y Biología Evolutiva, Museo Nacional de Ciencias Naturales, MNCN-CSICJosé Gutiérrez Abascal 2, 28006, Madrid, Spain; 4 Department of Biomedicine, University of TexasOne West University Blvd., Brownsville, Texas, 78520; 5 Department of Biological Sciences, Florida International UniversityMiami, Florida, 33199; 6 USDA-ARS-SHRS, National Germplasm RepositoryMiami, Florida, 33158; 7 Department of Biology, University of Puerto Rico-BayamonParque Industrial Minillas Carr 174, Bayamón, Puerto Rico, 00959-1911; 8 Departamento de Biología, Sección de Genética, Universidad del ValleAA 25360, Cali, Colombia; 9 Patricia and Phillip Frost Museum of Science, 3280 South Miami AvenueMiami, Florida

**Keywords:** *Avicennia germinans*, bottleneck, climate change, comparative phylogeography, gene flow, last glacial maximum, mangroves, Neotropics, population genetic structure, *Rhizophora mangle*

## Abstract

Comparative phylogeography offers a unique opportunity to understand the interplay between past environmental events and life-history traits on diversification of unrelated but co-distributed species. Here, we examined the effects of the quaternary climate fluctuations and palaeomarine currents and present-day marine currents on the extant patterns of genetic diversity in the two most conspicuous mangrove species of the Neotropics. The black (*Avicennia germinans,* Avicenniaceae) and the red (*Rhizophora mangle*, Rhizophoraceae) mangroves have similar geographic ranges but are very distantly related and show striking differences on their life-history traits. We sampled 18 Atlantic and 26 Pacific locations for *A. germinans* (*N* = 292) and *R. mangle* (*N* = 422). We performed coalescence simulations using microsatellite diversity to test for evidence of population change associated with quaternary climate fluctuations. In addition, we examined whether patterns of genetic variation were consistent with the directions of major marine (historical and present day) currents in the region. Our demographic analysis was grounded within a phylogeographic framework provided by the sequence analysis of two chloroplasts and one flanking microsatellite region in a subsample of individuals. The two mangrove species shared similar biogeographic histories including: (1) strong genetic breaks between Atlantic and Pacific ocean basins associated with the final closure of the Central American Isthmus (CAI), (2) evidence for simultaneous population declines between the mid-Pleistocene and early Holocene, (3) asymmetric historical migration with higher gene flow from the Atlantic to the Pacific oceans following the direction of the palaeomarine current, and (4) contemporary gene flow between West Africa and South America following the major Atlantic Ocean currents. Despite the remarkable differences in life-history traits of mangrove species, which should have had a strong influence on seed dispersal capability and, thus, population connectivity, we found that vicariant events, climate fluctuations and marine currents have shaped the distribution of genetic diversity in strikingly similar ways.

## Introduction

Mangrove forests are communities of trees and woody shrubs localized in intertidal zones of tropical and subtropical river deltas, lagoons, and estuarine coastal systems worldwide. These forests are composed of phylogenetically unrelated species, each of which has adapted to live at transition zones between land and sea. The main adaptations include tolerance to anoxic, saline and unstable soils, and aquatic seed dispersal (i.e., hydrochory) (Tomlinson 1986). The global distribution patterns of the different species that comprise mangrove forests are the result of a complex interplay among physiological constraints, dispersal and large-scale geological and climate events. Although adaptation to different habitats shapes local distributional patterns, it is likely that the combination of dispersal and historical perturbations has a predominate role in determining the overall distribution patterns (Duke 1995).

The comparison of phylogeographic patterns across nonrelated species that occupy similar ecological niches offers an exceptional opportunity to explore this interplay between historical and contemporary forces in shaping ecological communities (McGovern et al. 2010). Here, we tested the relative importance of these factors on the demographic history and population structure of the two most conspicuous co-distributed mangrove species in the Neotropics, the black mangrove, *Avicennia germinans* (Avicenniaceae), and the red mangrove, *Rhizophora mangle* (Rhizophoraceae). Although their ranges are largely overlapping, the two species differ markedly in major life-history characteristics including seed viability in salt water (3 months vs. 1 year), pollination syndrome (ambophilous vs. entomophilous), and seed shape (round vs. oblong), which may have an important effect on population connectivity (Sánchez-Nuñez and Mancera-Pineda 2012, Rabinowitz 1978). Nowadays, the two species are widely distributed on the Pacific and Atlantic coasts of tropical and subtropical regions of North, Central, and South America, and the range of both species extends to the west coast of Africa (Tomlinson 1986). Despite the large current distribution, Woodroffe and Grindrod (1991) speculated based on several lines of evidence, including some palynological data, that all species of the neotropical mangrove communities, including *A. germinans* and *R. mangle,* may have suffered several range contractions and expansions during the last glacial maximum (LGM). If correct, then these historical contractions should leave a strong imprint on the distribution of genetic variation among populations of the two species that we can test with molecular data.

We generated a multilocus genetic dataset composed of highly variable microsatellite, 2 chloroplast, and 1 anonymous nuclear loci and used these data to address three interrelated questions: (1) Is there genetic evidence of simultaneous population declines associated with quaternary climate changes? (2) Does the distribution of genetic variation follow the direction of palaeomarine currents or reflect contemporary marine currents? and (3) Does the current population genetic structure at large spatial scales reflect differences in the amount of time seeds remain viable in salt water?

## Materials and Methods

### Sampling localities

We sampled 292 individuals of *A. germinans* from 12 sites in the Atlantic Ocean (*N* = 208) and five in the Pacific Ocean (*N* = 84), and 422 individuals of *R. mangle* from 17 sites in the Atlantic Ocean (*N* = 269) and nine in the Pacific Ocean (*N* = 153) (see Appendix S1). Genomic DNA was extracted from ground leaf tissue using the DNeasy 96 plant kit (Qiagen, Valencia, CA) according to the manufacturer's protocol. All data, with the exception of those from individuals collected in Panama (Cerón-Souza et al. 2012), have not been previously reported.

### Microsatellite analysis

PCR conditions for 13 microsatellite loci in *A. germinans* (AgD13, AgT4, CA001, CA002, CT003, CTT001, GT003 CAT004, ACT004, GA003, and GT006) and six microsatellite loci in *R. mangle* (RM7, RM11, RM19, RM21, RM36, and RM46) followed previously described protocols (Cerón-Souza et al. 2012). For each sampling locality, we calculated the number of alleles per locus, the number of private alleles per locus, observed heterozygosity (*H*_*o*_), expected heterozygosity (*H*_*e*_), and the fixation index (*F*_IS_) for each species using GenAlEx 6.0 (Peakall and Smouse 2006). We also tested for deviation from Hardy–Weinberg equilibrium (HWE) and linkage equilibrium (LE) at each locus with Genepop 3.4 (Raymond and Rousset 1995). Finally, we assessed the quality of the data with respect to the presence of null alleles and scoring issues (using Microcheker 2.2.3, Van Oosterhout et al. 2004) and with the statistical power to detect genetic differentiation (using Powsim 4.0, Ryman and Palm 2006).

We computed AMOVA estimates and pairwise *F*_ST_ and *R*_ST_ estimators across sampling locations using Arlequin 3.1 (Excoffier et al. 2005) after 10,000 permutations. One (i.e., all sampling localities) and two (i.e., Atlantic vs. Pacific sites) group analyses were performed. We used FreeNA (Chapuis and Estoup 2007) to compare the pairwise results obtained at the one group level of structure, using the ENA method to correct the bias introduced by the presence of null alleles in the *F*_ST_ estimation after 10,000 replicates. In addition, we studied the relative importance of genetic drift versus mutation on the observed patterns of genetic variation in *A. germinans* and *R. mangle* for each locus after 10,000 random permutations with SPAGeDi 1.2 (Hardy and Vekemans 2002; Hardy et al. 2003). Finally, we tested the hypothesis of isolation-by-distance (IBD) across sampling localities with a linear stepping stone model of migration (Rousset 1997) using a Mantel test (Mantel 1967) after 10,000 permutations with GenAlEx 6.0 (Peakall and Smouse 2006).

We used Structure 2.3.2 (Pritchard et al. 2000; Falush et al. 2003, 2007) to determine the level of genetic structure for each mangrove species. For this analysis, we assumed an admixed model and a uniform prior probability of the number of populations, *K*. Ten independent runs were performed with 500,000 Markov chain Monte Carlo (MCMC) replicates after a burn-in of 50,000 replicates using a model of correlated allele frequencies and without information of sampling locations. Analyses were performed over all sampling locations (i.e., Neotropics) and within each ocean basin (i.e., Atlantic and Pacific). The best *K* and the Δ*K* (Evanno et al. 2005) for each analysis was evaluated in a range from the lower bound *K* = 1 to the upper bound *K* = 1 + [*n*^0.3^] (Bozdogan 1993), where *n* is the sample size of each dataset. For each distinctive cluster inferred by Structure, we used the program Migrate 3.2.6 (Beerli and Felsenstein 1999, 2001) to estimate historical effective population size Θ (defined as Θ = 4*N*_*e*_*μ*) and historical migration rates (*M*). To obtain an unscaled *N*_*e*_, we used mutation rates ranging from 10^−2^ to 10^−3^, as assumed for microsatellite loci in plants (Udupa and Baum 2001).

Based on the estimates of historical migration rates (*M*) obtained, we tested different models of gene flow within and between the Atlantic and Pacific oceans, using a Bayes factor approach (Beerli 2006; Beerli and Palczewski 2010). We conducted the analyses over two sets of data: the first one with microsatellite data structured according to geographical regions and the second set of data structured into two groups equivalent to the Atlantic and Pacific oceans. Unfortunately, the analysis with the first dataset did not return reliable confidence intervals using profile likelihoods even for very long runs (data not presented). Between oceans, three different models were evaluated for the two species: (1) a panmictic model with one population size (Atlantic and Pacific oceans), (2) two population sizes and one migration rate to the Pacific Ocean, and (3) two population sizes and one migration rate to the Atlantic Ocean. The models were compared using Bézier thermodynamic integration (Beerli and Palczewski 2010), and their marginal likelihoods were then used to calculate Bayes factors and model probabilities (Kass and Raftery 1995). We assumed a Brownian motion mutation model with constant mutation across all loci. MCMC consisted of ten short chain samplings (of 50,000 trees) and three long chain samplings (of 500,000 trees) using an adaptive heating scheme.

A Bayesian approach was also used to determine contemporary gene flow among *A. germinans* and *R. mangle* populations within each ocean as implemented in BayesAss 1.3 (Wilson and Rannala 2003). Since the software allows the estimation of recent migration rates (within the past three generations), we performed the analysis only within each ocean to establish a more reduced temporal scale (Montes et al. 2012; Coates and Stallard 2013). Samples were run for 3 × 10^7^ generations with a “burn-in” period of 10^6^ generations and sampled every 2000 generations. Two independent runs were performed to check for convergence of results.

Finally, we assessed the scale and timing of demographic changes using the Bayesian coalescent model-based approach implemented in Msvar 1.3 (Beaumont 1999; Storz and Beaumont 2002). Msvar uses MCMC simulations to estimate the posterior probability distribution of a set of population log-normal parameters: current (*N*_0_) and ancestral (*N*_1_) effective population size, *μ* (mutation rate), and *X*_*a*_ (time interval in years), where *X*_*a*_ = g (generation length, in years per generation) × *T*_*a*_ (number of generations since expansion/decline). A hierarchical model (that allows demographic and mutational parameters to vary among loci), a strict stepwise mutation model, and an exponential change in size of populations were used for the analyses (Storz and Beaumont 2002). Based on aging techniques adapted for *Rhizophora*, *Avicennia,* and *Sonneratia*, a conservative generation time ranging between 10 and 40 years has been estimated (Polidoro et al. 2010). Mangroves can survive up to 100 years (Lopez-Hoffman et al. 2007). Thus, a generation time of 100 years was also used to generate broad confidence intervals around the estimates of absolute time (Jones et al. 2013). Five MCMC chains were conducted for 24 million generations with 50,000 thinning intervals using the same uninformative log-normal distribution of starting points (Table 1). We assessed the convergence among five chains using the Gelman–Rubin statistics (Gelman and Rubin 1992; Brooks and Gelman 1998) calculated in the R package BOA (Smith 2007). When convergence was reached, we estimated the mean, standard deviation, and 95% highest posterior density (HPD) for each of the parameters using a burn-in of half of the five merged chains, also with the R package BOA (Smith 2007).

**Table 1:** Lognormal priors (means/standard deviations, SD) and hyperpriors (for means and variances) used for Msvar analysis performed on *Rhizophora mangle* and *Avicennia germinans*. *N*_*0*_ and *N*_*1*_ are current and ancestral effective population sizes, respectively, *μ* is the mutation rate, and Ta is the number of generations since expansion/decline

Parameter	Log_10_ Mean/SD	Hyperpriors	Log_10_ Mean/SD/hyperpriors of mean/SD
*N*_*0*_	*3*/*3*	*αN*_*0*_*σN_0_ βN_0_ τN*_*0*_	*6/2/0/0.5*
*N*_*1*_	*3*/*3*	*αN*_*1*_*σN_1_ βN_1_ τN*_*1*_	6/2/0/0.5
*μ*	–3/1	*αμ σμ βμ τμ*	–3/0.25/0/0.5
Ta	4/3	*α*Ta *σ*Ta *β*Ta *τ*Ta	8/2/0/0.5

### Chloroplast and nuclear sequences analyses

For a subset of individuals, we also analyzed DNA sequence variation at two chloroplast (cpDNA) noncoding loci, *atpI-atpH* and *psbJ-petA* (Shaw et al. 2007) and at the Agerm-CT004 nuclear flanking microsatellite region (FMR) loci for *A. germinans* and the RM11 loci for *R. mangle* (Rosero-Galindo et al. 2002). The amplification and sequencing procedures for cpDNA and the RM11 followed previously established protocols (Cerón-Souza et al. 2010, 2012). In *A. germinans*, the primers used to amplify the CT004 FMR were as follows: F: 5′ CAATTCCTTGGGTAATTCTTGG and R: 5′ GGCAAGGTTGCTGGAATATG. We calculated nucleotide diversity (*π*) and haplotype diversity using DnaSP v4.10.3 (Rozas et al. 2003). We also constructed a median-joining network (Bandelt et al. 1999) for each nuclear FMR and the two cpDNA gene regions together using Network v4.5.1.0 (http://www.fluxus-engineering.com/).

## Results

### Microsatellite diversity and population genetic structure

Microsatellite polymorphism and genetic diversity were high for both species (Appendix S1). Mean observed and expected heterozygosities were *Ho* = 0.40 ± 0.02 SE and *H*_E_ 0.42 ± 0.02 SE for *A. germinans* and *Ho* = 0.36 ± 0.02 SE and *H*_E_ = 0.38 ± 0.03 SE for *R. mangle*. There was no compelling evidence of genotyping errors. However, certain loci in some locations showed strong evidence of null alleles with frequencies that ranged from 0.11 to 0.79 in *A. germinans* and from 0.07 to 0.71 in *R. mangle* (Appendix S1). The presence of null alleles may partially explain the significant deviation from HWE that we observed in over half of the sampling localities for *A. germinans* and *R. mangle*. For example, significant deviations of *F*_IS_ from zero due to an excess of homozygotes were detected at nine localities of *A. germinans* and 11 localities of *R. mangle* (Appendix S1). In addition, most of the localities with HW deviations in *R. mangle* were located in the Pacific Ocean, where we have evidence of current introgressive hybridization with its sister species *Rhizophora racemosa* (Cerón-Souza et al. 2010). With the exception of the Panama–Montijo Gulf site, there was no deviation from linkage equilibrium (LE) in either species.

All loci used had a high power to detect *F*_ST_ values as low as 0.005 with a probability of 100% for *A. germinans* and 95% for *R. mangle*. Hierarchical AMOVA revealed highly significant genetic structuring (overall *F*_ST_ = 0.35, *R*_ST_ = 0.46 values for *A. germinans* and *F*_ST_ = 0.45, *R*_ST_ = 0.37 values for *R. mangle,* Table 2). The 28–44% variance in *A. germinans* and 14–38% in *R. mangle*, depending on the mutation model assumed (i.e., IAM or SMM), was associated with the CAI barrier (Table 2). Pairwise estimates among sampling localities showed a high number of significant comparisons (99% and 79% in *A. germinans* and 98% and 87% in *R. mangle,* depending on the mutation model used) (Appendix S2). Genetic drift rather than mutation was the main cause of genetic differentiation across all localities in both mangrove species (i.e., *R*_ST_ = *ρR*_ST_), with some exceptions (e.g., French Guiana and Trinidad vs. nearby localities in *A. germinans*, see Appendix S2). We did not detect any strong isolation by distance in either species.

**Table 2:** Analysis of molecular variance (AMOVA) of *Avicennia germinans* and *Rhizophora mangle* at two different levels of structure: one group (i.e., all sampling localities) and two groups (i.e., Atlantic and Pacific Ocean populations on both sides of the Central American Isthmus). Bold *P* numbers are significant values (*P*<0.05)

	Weir and Cockerham (1984)				Slatkin (1995)			
Species	Variance	% Total	*F* statistics	*P*	Variance	% Total	*R* statistics	*P*
*Avicennia germinans*								
One group								
Among sampling localities	1.411	35.03	*F*_ST_ = 0.350	**0.000**	456.209	46.23	*R*_ST_ = 0.462	**0.000**
Within sampling localities	2.616	64.97			530.527	53.77		
Two groups: Atlantic vs. Pacific Ocean								
Among groups	0.695	28.07	*F*_CT_ = 0.281	**0.000**	123.491	44.01	*R*_CT_ = 0.440	**0.000**
Within groups	0.501	20.26	*F*sc = 0.282	**0.000**	68.414	24.38	*R*sc = 0.435	**0.000**
Within sampling localities	1.278	51.67	*F*_ST_ = 0.483	**0.000**	88.708	31.61	*R*_ST_ = 0.684	**0.000**
*Rhizophora mangle*								
One group								
Among sampling localities	0.870	44.59	*F*_ST_ = 0.446	**0.000**	22.257	36.48	*R*_ST_ = 0.365	**0.000**
Within sampling localities	1.081	55.41			38.749	63.52		
Two groups: Atlantic vs. Pacific Ocean								
Among groups	0.632	38.09	*F*_CT_ = 0.381	**0.000**	8.146	14.66	*R*_CT_ = 0.147	**0.001**
Within groups	0.301	18.15	*F*sc = 0.293	**0.000**	12.155	21.88	*R*sc = 0.256	**0.000**
Within sampling localities	0.726	43.76	*F*_ST_ = 0.562	**0.000**	35.250	63.45	*R*_ST_ = 0.366	**0.000**

**Figure 1:**
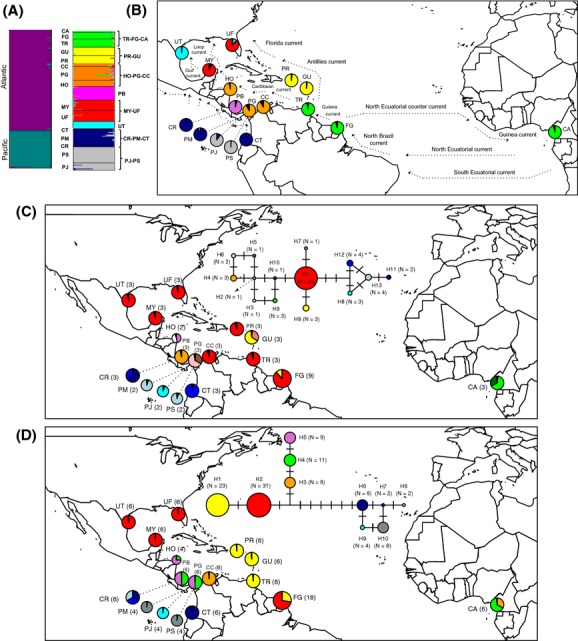
Genetic structure across 17 sites of *Avicennia germinans* in the Neotropics using nuclear microsatellite polymorphisms (A and B) and median-joining networks based on haplotype polymorphisms for two chloroplast genes (C) and one flanking microsatellite region (FMR) (D). (A) Structure analysis based on microsatellite polymorphisms for the entire set of sampling locations (i.e., Neotropics) (best *K* = 2) and by ocean basin (i.e., Atlantic, best *K* = 6 and Pacific, best *K* = 2). (B) The map shows the proportion of assignment for each of the sampling locations to inferred clusters within each ocean basin and the main ocean currents that surround each sampling locality (summarized from Gordon 1967, Kessler 2006, Lee *et al*. 1996, Oey *et al*. 1985, Richardson and Walsh 1986, Schott and Zantopp 1985, Stramma and Schott 1999 and Wüst 1964). (C) The inferred median-joining networks for cpDNA loci (*atpI-atpH* and *psbJ-petA*) and (D) for the FMR region (CT004). For each network, the proportion of haplotypes found in each sampling location and the number of sequences analyzed (in parenthesis) by sampling locality is also indicated (see Appendix S1 for the acronyms of each sampling location).

**Figure 2:**
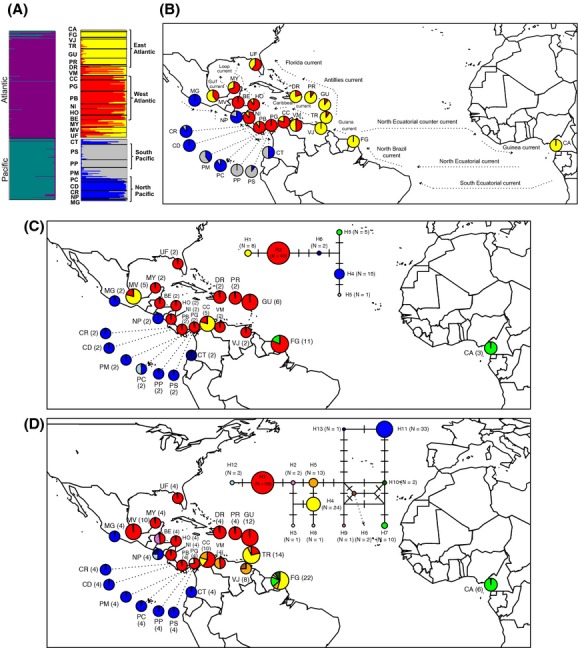
Genetic structure across 26 sites of *Rhizophora mangle* in the Neotropics using nuclear microsatellite polymorphisms (A and B) and median-joining networks based on haplotype polymorphisms for two chloroplast genes (C) and one flanking microsatellite region (FMR) (D). (A) Structure analysis based on microsatellite polymorphism for the entire set of sampling locations (i.e., Neotropics) (best *K* = 2) and grouping of the sampling locations by ocean basin inferred (i.e., Atlantic, best *K* = 2 and Pacific, best *K* = 2). (B) The map shows the proportion of assignment for each of the sampling locations to inferred clusters within each ocean basin and the main ocean currents that surround each sampling locality. (summarized from Gordon 1967, Kessler 2006, Lee *et al*. 1996, Oey *et al*. 1985, Richardson and Walsh 1986, Schott and Zantopp 1985, Stramma and Schott 1999 and Wüst 1964) (C) The inferred median-joining networks for cpDNA loci (*atpI-atpH* and *psbJ-petA*) and (D) for the FMR regions (RM11). For each network, the proportion of haplotypes found in each sampling location and the number of sequences analyzed (in parenthesis) by sampling locality is also indicated (see Appendix S1 for the acronyms of each sampling locations).

When all sampling locations were tested together, Structure analyses showed a highest posterior likelihood of *K* = 2 for both species. The two inferred clusters clearly corresponded with the Atlantic and the Pacific ocean basins, highlighting the importance of the CAI as a barrier to gene flow for the mangrove species (Figs1A,B and 2A,B). Analyses performed on samples from each ocean basin also revealed genetic structure mainly associated with the geographic distribution of the individuals. Within the Atlantic Ocean, *K* was six for *A. germinans* and two for *R. mangle*, whereas within the Pacific Ocean, *K* was equal to two for both species.

Most individuals were assigned to their respective clusters with a high average proportion of membership (Q), ranging from 88% to 90% for *A. germinans* and 79% to 83% for *R. mangle*. However, some *R. mangle* individuals sampled at UF, MV, MY, VM, PM, and CT showed high degrees of admixture with lower assignment probability to the two clusters (Q > 36%) (Fig. 2A). Both species showed evidence of trans-Atlantic gene flow where the West African locality (Cameroon) was assigned to the same cluster as American clusters (i.e., FG-TR-CA in *A. germinans* and East Atlantic in *R. mangle*).

### Historical effective population size based on microsatellite diversity

**Figure 3:**
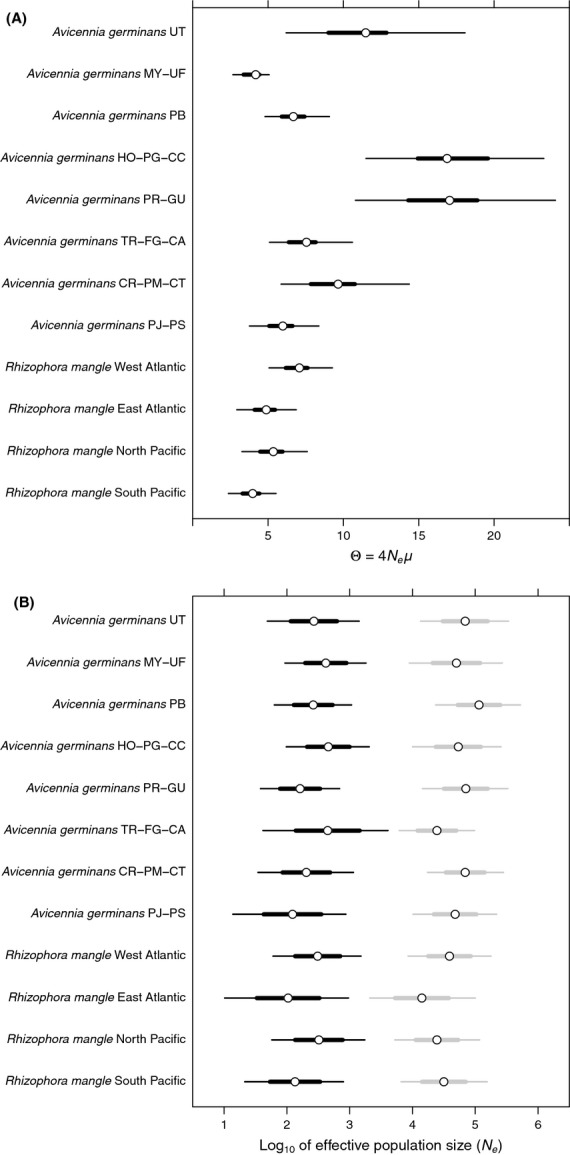
Historical demography of eight clusters of *Avicennia germinans* and four clusters of *Rhizophora mangle* inferred by Structure. (A) Theta values (Θ = 4*N*_*e*_*μ*) estimated with Migrate and (B) the log_10_ of current (*N*_0_) (in black) and ancient (*N*_1_) (in gray) effective population size (*N*_*e*_) estimated with Msvar. The circles are mean estimates; the thick hash marks are from the 2.5% to the 97.5% percentile of theta (A) and the standard deviations of *N*_*e*_ (B). The thin hash mark indicates the 25–75 percentile of theta (A) and the 95% highest posterior density in *N*_*e*_ (B). Names of Structure clusters for each species are the same as in Figures1 and 2. All are estimated with Msvar showing a 97.5% quantile of the Gelman–Rubin statistics <1.02.

The MIGRATE estimates of the population size parameter (Θ) ranged from 4.18 to 17.05 for *A. germinans* and 3.98 to 7.08 for *R. mangle* (Fig. 3A). Long-term transformed *Ne* values (assuming a *μ* of 10^−2^ and 10^−3^) (Udupa and Baum 2001) ranged from 575 to 2,321 for *A. germinans* and from 548 to 974 for *R. mangle*, respectively. We did not find significant differences (*P* < 0.05) in Θ and *N*_*e*_ among the two mangrove species. However, two *A. germinans* clusters localized on the Atlantic coast of Central America and northern South America (HO-PG-CC) and in the Antilles islands (PR-GU) showed higher Θ and *N*_*e*_ values compared with all four *R. mangle* clusters (Fig. 3A).

### Historical and current migration patterns based on microsatellite diversity

The comparison of the three historical gene flow hypotheses revealed that for both species, the second model (i.e., Atlantic Ocean as a source and the Pacific Ocean receiving migrants) was favored over the rest of the models tested (Table 3). The current migration rates inferred with BayesAss within each ocean were in general low (i.e., *M* < 0.1). However, in the sites surrounding the Antilles Islands within Caribbean, we found asymmetrical patterns of migration with values of *M* > 0.1 in both species that were congruent with the direction of marine currents (Fig2B and 3B). This includes migration from Guadeloupe to Puerto Rico following the Antillean marine current (Lee *et al*. 1996) and migration from Colombian and Panamanian sites toward Honduras, Nicaragua, Mexico, and Florida following the Caribbean marine current (Gordon 1967). Within the Pacific Ocean basin, we found bidirectional migration rates of *M* > 0.1 restricted to neighboring localities within Panama and between Panama and Costa Rica. Again, dispersal was congruent with the short and seasonal currents, and the permanent eddies that characterize the marine circulation patterns of the eastern tropical Pacific (Kessler 2006) (Appendix S4).

**Table 3:** Comparison of three models of gene flow for *A. germinans* and *R. mangle* samples across the Atlantic (Atl) and Pacific (Pac) oceans

Species	Model	Ln mL	Ln Bayes factor	Model probability
*Avicennia germinans*	1) Panmictic	−829,696.46	−455,410.88	0.00
	2) Directional (Atl to Pac)	−601,991.02	0.00	1.00
	3) Directional (Pac to Atl)	−665,653.97	−127,325.90	0.00
*Rhizophora mangle*	1) Panmictic	−252,114.12	−206,076.94	0.00
	2) Directional (Atl to Pac)	−149,075.65	0.00	1.00
	3) Directional (Pac to Atl)	−153,737.28	−9323.26	0.00

### Changes in effective population size over time based on microsatellite diversity

**Figure 4:**
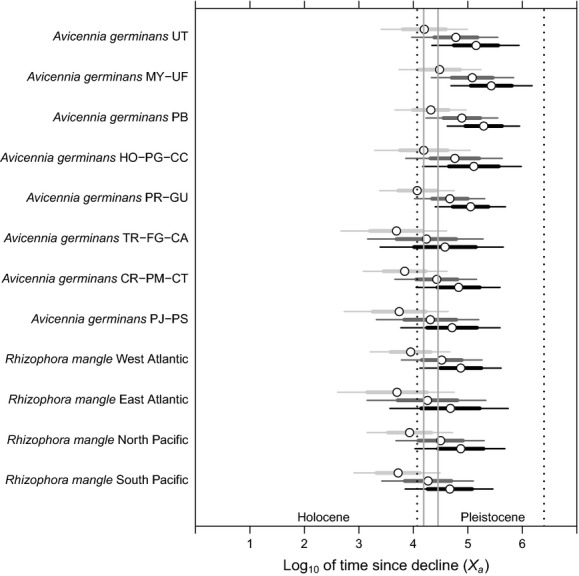
Posterior distribution of the time since population decline (*X*_*a*_) estimated in Msvar for eight clusters in *Avicennia germinans* and four clusters in *Rhizophora mangle* inferred with Structure, assuming 10 years (light gray), 40 years (dark gray), and 100 years (black) of generation time (g). The large circles are mean estimates, the thick hash marks are the standard deviations, and the thin hash marks are the 95% highest posterior density, respectively. The two gray vertical lines represent the last glacial maximum (LGM) interval for the Northern Hemisphere (17,800–26,500 years). The two black dotted lines represent the beginning of the Pleistocene (2,588,000 years) and the beginning of the Holocene (11,700 years). The names of Structure clusters for each species are the same as in Figures1 and 2. All estimates showing a 97.5% quantile of the Gelman–Rubin statistics <1.02.

The Msvar analysis showed clear evidence of population declines across *A. germinans* and *R. mangle* mangrove populations. Current population sizes (*N*_*0*_) are on average two hundred times smaller than ancestral effective population sizes (*N*_*1*_) (Fig. 3B). The mean estimates of time since this decline (*X*_*a*_) were placed from 5000 to 30,200 years (at g = 10), from 17,300 to 120,200 years (at g = 40), and from 38,000 to 297,000 years (at g = 100) across all clusters of both species. Although some *A. germinans* clusters in the Caribbean Sea (i.e., UT, MY-UF, HO-PG-CC, PB, and PR-GU Structure clusters) showed older X_*a*_ values, all 95% HPD estimates within and between the two species indicated that population declines of *A. germinans* and *R. mangle* occurred during the mid-Pleistocene to early Holocene (Fig. 4).

### Chloroplast and nuclear haplotype networks

The neighbor-joining network subdivided the cpDNA and FMR haplotypes into two main groups supporting the strong phylogeographic pattern associated with the closure of the CAI also observed with microsatellite polymorphisms (Figs1C,D and 2C,D). In *A. germinans,* the two distinctive haplogroups were separated by two and six substitutions in the cpDNA and FMR genomes, respectively. In addition, within the Atlantic Ocean, the nuclear FMR shared haplotypes (i.e., H3 and H4) between West Africa and Central and South America suggesting trans-Atlantic gene flow (Fig. 1C,D). In the case of *R. mangle,* the separation of cpDNA haplotypes across the CAI was less pronounced than in *A. germinans* due to the close relatedness of the H3 haplotype found in Atlantic samples to two haplotypes found in Pacific samples (i.e., H4 and H6) (Fig. 2C). Similarly, in the FMR network, haplotype H12 from the Pacific Ocean (Nicaragua) was more closely related to Atlantic H1 than any other Pacific haplotype (Fig. 2D). Both genomes of *R. mangle* also support the observed trans-Atlantic gene flow, since shared haplotypes between West Africa (Cameroon) and South America (French Guiana) were found (i.e., H3 in cpDNA and H7 in FMR) (Fig. 2C,D). Haplotype diversity and the number of haplotypes for each gene and species are indicated in Appendix S3.

## Discussion

Despite striking differences in life-history traits between *A. germinans* and *R. mangle*, their extant genetic diversity has been similarly shaped by a combination of major geological events, quaternary climate fluctuations, and the contemporary and palaeomarine currents. Our data reaffirm the strong effect of the Central American Isthmus (CAI) closure on the diversification history of neotropical mangroves (Nettel and Dodd 2007; Cerón-Souza et al. 2010, 2012; Takayama et al. 2013). The CAI clearly divided current Pacific and Atlantic populations of both *Avicennia* and *Rhizophora* and, as a consequence, Atlantic and Pacific ocean mangrove populations should be considered independent units that have evolved separately since the Miocene–Pliocene (Coates and Obando 1996; Montes et al. 2012).

In addition to the genetic differences among populations separated by the CAI, there was a strong signal of subsequent population bottleneck in all Atlantic and Pacific populations that we examined. The relative timing of this bottleneck was remarkably consistent across distinct populations, and nearly, all estimates placed the date around the last glacial maximum (LGM) (c.a. 28,300–15,440 years BP, revised in Shakun & Carlson, 2010) (Fig. 4). These data are consistent with evidence for population declines during this time from the palynological and peat stratigraphy data for mangroves (Ellison and Stoddart 1991). Lower sea levels likely physically reduced suitable mangrove habitat across the region (Donoghue 2011). At the same time, the cooler ocean temperatures likely pushed populations further toward the equator. Mangroves are very sensitive to cool temperatures (Kao et al. 2004; Stuart et al. 2007), to the point that the present day distribution of mangroves is thought to reflect physiological rather than dispersal limitations (Duke et al. 1998; Krauss et al. 2008). Our very low estimates of effective population size within contemporary mangrove populations support this hypothesis. Thus, although *Avicennia* and *Rhizophora* have been present in the Neotropics for the last 16 (Miocene) and 40 Ma (Eocene) respectively, the current genetic diversity within both species is a legacy of the quaternary fluctuations in population size and range (Hewitt 2000).

The low genetic diversity in chloroplast and nuclear regions and the fixed haplotypes found in *A. germinans* and *R. mangle* across hundreds of kilometers on both sides of the CAI are likely caused by recent range expansion during the Holocene. Similar conclusions have been reached previously based on a combination of fossil (Ellison 1993; Jaramillo and Bayona 2000) and molecular evidence (Nettel and Dodd 2007; Pil et al. 2011; Sandoval-Castro et al. 2012). These studies have had either a much narrower geographic scope or fewer species, but similarly show evidence for a quaternary reduction in population size and Holocene expansions. Together, these data reinforce the idea that current mangrove distribution and genetic structure are probably more correlated with habitat availability and local history of extinction/expansion than dispersal capacity (Ellison 1993).

### The effect of marine currents

The direction of main marine currents, both historical and contemporary, has acted with past climate to shape population genetic structure in mangroves. Marine palaeo-oceanographic models support the existence of a strong current through the Central American seaway from the Atlantic toward the Pacific oceans during the Miocene–Pleistocene (i.e., before the final closure of the CAI) (Iturralde-Vinent 2006). Consistent with this westward flowing current, estimates of migration best fit an asymmetrical gene flow model with higher rates of historical migration from the Atlantic to the Pacific oceans. Likewise, our genetic data suggest a strong role of contemporary currents in the distribution of genetic variation, similar to recent reports for *Rhizophora mucronata* in South-East Asia (Wee et al., 2014). There was a strong genetic affinity between African and eastern South American populations of both species consistent with the main currents, the North Equatorial Current (NEC), the South Equatorial Current (SEC), and the North Equatorial Counter Current (NECC), which connects America with West Africa (Richardson and Walsh 1986, Stramma and Schott 1999) (Figs1B and 2B). These currents were established following the breakup of West Gondwana and have maintained the same direction since the final closure of the CAI (Parrish 1993; Renner 2004). Large floating objects with wind-exposed surfaces can cross the Atlantic Ocean in <2 weeks (Renner 2004), a timeframe that is far shorter than the seed viability in saltwater for both *A. germinans* (c.a. 90 days) and *R. mangle* (c.a. 360 days) (Rabinowitz 1978).

Similarly, genetic patterns within the West Atlantic and in the Pacific reflected the prevailing current patterns. For example, we found high migration (*M* > 0.1) rates from Guadeloupe toward Puerto Rico in both species following the direction of the Antilles current (Lee *et al.* 1996). There was also strong evidence for directional migration from sites in Colombia and Panama toward Honduras, Nicaragua, and Mexico following the direction of the Caribbean current (Gordon 1967). In the eastern tropical Pacific, high and bidirectional migration (*M* > 0.1) was evident in neighboring localities within Panama and between Panama and Costa Rica of both species. This pattern is consistent with short-distance currents, seasonal changes, and permanent eddies that characterize ocean circulation patterns in the east tropical Pacific. In October–December, main currents between northern Colombia, Panama, and Costa Rica follow a highly dynamic northwesterly direction. In comparison, in January–March, the dynamic of these currents decreases and although the main northwesterly direction is maintained, there are also opposing currents moving in the eastward direction (Kessler 2006).

### The effect of life-history traits

Our comparative approach reinforces the clear differences between the two mangrove species in levels of population subdivision and historical demography. *R. mangle* populations exhibit much less genetic structuring and, consequently higher gene flow, relative to populations of *A. germinans*. These differences are consistent with the difference in seed viability in saltwater (90 days and 360 days) and pollination syndrome (ambophilous for *R. mangle* and entomophilous for *A. germinans*) that characterize the two species (Cerón-Souza et al. 2012). Furthermore, we found that the historical population size of *A. germinans* in the Caribbean Sea clusters (HO-PG-CC and PR-GU) was higher than all four *R. mangle* clusters (Fig. 3A). This finding contradicts the striking differences in population density observed in the Caribbean and Central America today (Lovelock et al., 2005) and leads us to question why current *A. germinans* populations are less dense and more fragmented than they were in the past.

One possible explanation for this shift in dominance is the remarkable ability for *R. mangle* to disperse its seeds over long distances relative to other neotropical mangrove species. Besides trans-oceanic dispersal of *R. mangle* reported in the Atlantic, genetic analysis in the Pacific basin supports long distance gene flow events that occurred from the East Pacific coast toward the Pacific islands, which has not been observed in other mangrove species (Takayama et al. 2013; Lo et al., 2014). The biogeographic analysis of *Rhizophora* species around the world suggests that modern geographic distribution and disjunctions of this genus are explained by both vicariance, but also by several unique trans-oceanic dispersal events (Lo et al., 2014). Thus, it is possible that *R. mangle's* remarkable seed viability in saltwater made it a superior competitor compared to other mangrove species during the Holocene when it recolonized new areas.

Alternatively, this shift in palaeo-ecological succession may be associated with local adaptation of *A. germinans* to cooler temperatures. Although current *A. germinans* populations have a much more fragmented distribution than *R. mangle* (Cerón-Souza et al. 2012), *A. germinans* extends north to the colder Gulf of Mexico coasts of Texas, Louisiana, and the extreme north of Florida where *R. mangle* is largely absent (Sherrod et al., 1986). Different experiments have demonstrated that *A. germinans* from these northern areas have a greater chilling tolerance, especially in early life-history stages (Markley et al., 1982; Pickens & Hester, 2011). Thus, it is possible that cooler temperatures favored the distribution and abundance of *A. germinans* in the past, when they dominated the landscape. Then, during the Holocene period when changes in sea level and precipitation patterns were accompanied with warmer temperatures, *Rhizophora* gradually replaced *Avicennia*. The analysis of fossilized pollen from Guyana, Surinam, and Colombia supports this scenario and suggests that this species succession pattern occurred similarly in distant regions in the tropical belt during the Holocene (Van der Hammen, 1974; Urrego et al., 2013).

### The effect of future climate change on mangroves

Mangrove forests are one of the most threatened ecosystems in the world (Valiela et al. 2001; Alongi 2002). Neotropical mangroves are being destroyed and fragmented at alarmingly high rates, mainly due to coastal development, aquaculture expansion, and overharvesting (Polidoro et al. 2010). Moreover, mangroves are extremely sensitive to climate change and sea-level fluctuations because of their localization in coastal areas at low altitudes (Alongi 2008, 2015). The current models of species and community distribution under climate change predict that neotropical mangroves, including those on the Atlantic and Pacific coasts of Central America and the Caribbean, must be classified as critically endangered (Polidoro et al. 2010; Record et al. 2013). These models predict that mangrove species (including both *A. germinans* and *R. mangle*) in neotropical regions will experience (1) a pole-ward shift under future climate scenarios, especially on North America (Alongi 2015), and (2) an overall contraction of species distributions and a severe decline in species richness in response to increasing temperatures and salinity and acidity of the soils as well as a decrease in precipitation (Record et al. 2013; Alongi 2015). This alarming prediction for neotropical mangrove areas based on species distribution and adjusted climate variables is remarkably similar to the bottleneck pattern that occurred in both species between the mid-Pleistocene and the early Holocene. Indeed, our historical demographic reconstruction based on molecular data suggests that, although *A. germinans* and *R. mangle* will likely survive global warming, the genetic diversity of both mangrove species will be critically eroded.

## Supporting Information

**Appendix S1**. Summary of genetic diversity measurements observed across 17 and 26 sampling localities of *Avicennia germinans* (13 loci) and *Rhizophora mangle* (six loci).

**Appendix S2**. Pairwise comparisons of *F*_ST_ (Weir & Cockerham 1984) (below the diagonal) and *R*_ST_ (Slatkin 1995) (above the diagonal) among 17 sampling localities of *Avicennia germinans* (13 loci) and 26 sampling localities of *Rhizophora mangle* (six loci) distributed in the Atlantic and Pacific Oceans.

**Appendix S3.** Descriptive statistics and Genbank accession numbers of sequences from two cpDNA (atpI-atpH and psbJ-petA that were analysed together) and one Flanking Microsatellite Region from *A. germinans* (*n* = 53) and *R. mangle* (*n* = 79) distributed in the Atlantic and Pacific Oceans. For each genome the number of variable sites including transitions (TA), transversions (TR) and the number of indels (in parenthesis) as well as the length of each indel in pb are shown. Also reported is nucleotide diversity (p), haplotype diversity (Hd) with their standard deviations (SD) in parenthesis and the number of haplotypes found (H).

**Appendix S4.** Pairwise comparisons of recent migration (m) rates among 17 sampling localities of *Avicennia germinans* (13 loci) and 26 sampling localities of *Rhizophora mangle* (six loci) within the Atlantic and Pacific Oceans inferred with BayesAss.
